# Dietary Malic Acid Supplementation Induces Skeletal Muscle Fiber-Type Transition of Weaned Piglets and Further Improves Meat Quality of Finishing Pigs

**DOI:** 10.3389/fnut.2021.825495

**Published:** 2022-01-25

**Authors:** Xin Zhang, Meixia Chen, Enfa Yan, Yubo Wang, Chenghong Ma, Pengguang Zhang, Jingdong Yin

**Affiliations:** ^1^State Key Laboratory of Animal Nutrition, College of Animal Science and Technology, China Agricultural University, Beijing, China; ^2^Institute of Animal Husbandry and Veterinary Medicine, Beijing Academy of Agriculture and Forestry Sciences, Beijing, China

**Keywords:** malic acid, skeletal muscle fiber-type transition, antioxidant capacity, weaned piglets, pork quality

## Abstract

The aim of this study was to investigate effects of dietary malic acid supplementation on skeletal muscle fiber-type transition during nursery period and the subsequent meat quality of finishing pigs. Results showed that malic acid supplementation for 28 days increased oxidative fiber percentage of weaned piglets, accompanied by the increased aerobic oxidation in serum and longissimus thoracis (LT) muscle. Additionally, activities of total antioxidant capacity and glutathione peroxidase in serum were increased. Moreover, dietary malic acid supplementation during nursery period tended to increase pH_24h_ and significantly decreased drip loss in LT muscle of finishing pigs. The content of total saturated fatty acid (SFA) and total monounsaturated fatty acid in LT muscle was significantly decreased, whereas the ratio of polyunsaturated fatty acid to SFA tended to increase. Together, dietary malic acid supplementation during nursery period can effectively increase antioxidant capacity and oxidative fibers percentage of weaned piglets, and further improve water holding capacity and nutritional values of pork in finishing pigs.

## Introduction

Pork is an important meat source for humans. According to a report by USDA (http://www.ers.usda.gov/topics), the worldwide pork consumption was ~100.9 million tons in 2019. However, with the advancement of animal genetics, nutrition, and management, excessive pursuit of higher growth rate and lean meat percentage of pigs has led to the deterioration of pork quality, such as lower early postmortem pH values, greater drip loss, and higher shear values (1).

Drip loss, a key parameter of water holding capacity of fresh meat (2), is critical for customer experience and meat industry profitability. In particular, it directly influences the appearance and sensory quality, and closely correlates with other meat quality parameters, such as pH value, meat color, and marbling (3, 4). Furthermore, the coefficient of variance was higher for drip loss than other pork quality parameters, and may enhance the adverse impact on further processing of the pork product (5). Of note, skeletal muscle is heterogeneous and composed of four fiber types, including type I, type IIa, type IIx, and type IIb based on the expression of myosin heavy chain (MyHC) isoforms and metabolic activity. Oxidative fibers (type I and type IIa) are characterized by smaller size, higher concentrations of myoglobin, and more mitochondria compared with glycolytic fibers (type IIx and type IIb) (6, 7). Growing evidence highlights that higher percentage of oxidative fibers leads to a lower degree of glycolysis and contributes to low drip loss, high pH value, and redness (8, 9). Based on this, nutritional strategies, such as the supplementation of natural phytochemicals (10, 11), have been applied to improve pork quality partly through influencing muscle fiber-type composition in finishing pigs. Of significant importance is that the wave of muscle fiber-type transition in pigs occurs from birth to 60 days of age, named window period (12–14). Therefore, more studies need to address the impact of muscle fiber-type transition during window period on pork quality.

Malic acid, an intermediate in the tricarboxylic acid (TCA) cycle, could participate in mitochondrial energy metabolism and has many applications in food, beverage, pharmaceutical, chemical, and medical industries (15). It has been reported that intestinal concentration of malic acid was positively correlated with the circulating cholesterol and low-density lipoprotein cholesterol (16). The negative correlation between muscle malic acid content and obesity was also observed in rabbits (17), suggesting that malic acid was involved in the development of metabolic syndrome. The activity of malate dehydrogenase (MDH), an essential enzyme for the conversion of malic acid to oxaloacetate, is typically used as a marker of skeletal muscle oxidative capacities. l-Malic acid was used as a component of organic acid complex to improve the growth performance of sucking piglets (18) and weaned piglets (19) in previous studies. However, the application of l-Malic acid in animal husbandry is still limited, and the effects of dietary supplementation of malic acid on skeletal muscle fiber-type transition and pork quality is unknown.

Consequently, the objective of this study is to assess the effects of dietary malic acid supplementation on skeletal muscle fiber-type transition of weaned piglets, and the subsequent meat quality of finishing pigs. This study provides the first evidence for malic acid as a promising candidate to reduce drip loss and improve the nutritional value of pork through inducing transition of skeletal muscle fiber-type in the window period.

## Materials and Methods

### Ethics Statement

Animal trials were conducted in accordance with the Guidelines for Care and Use of Laboratory Animals of China Agricultural University (ID: SKLAB-B-2010-003).

### Experimental Design and Sample Collection

In Experiment 1, a total of 192 28-day old Duroc × Landrace × Large White crossbred castrated male piglets with an average body weight (BW) of 9.12 kg were assigned to receive four dietary treatments in a randomized complete block design based on the initial BW, with six replicates (pens) per treatment and eight piglets per pen. The basal diet was formulated to meet the National Research Council (NRC, 2012) nutrient requirements for pigs of 11–25 kg BW without supplementation of any antibiotic additives. Four dietary treatments included basal diet (control) and a basal diet supplemented with 2.5, 5, or 10 g malic acid complex kg^−1^ diet, respectively. Malic acid complex was obtained from Anhui Sealong Biotechnology Co., Ltd. (Bengbu, Anhui Province, China), which was composed of 20% l-malic acid and 80% carrier (zeolite powder). The ingredient composition and nutrient level of basal diet are shown in Supplementary Table 1. All piglets were fed *ad libitum* and free access to clean drinking-water for 28 days.

On days 14 and, feed intake and BW were recorded to calculate the average daily feed intake (ADFI) and average daily gain (ADG). The ratio of ADG to ADFI was used as feed conversion ratio (FCR). Furthermore, blood samples were also collected on day 14 and day 28 from the precaval vein of piglets with average BW of each pen (*n* = 6) after overnight starvation for 16 h. Serum was separated and stored at −20°C for further analysis.

At the end of Experiment 1, pigs with the average final BW from six pens of each group (*n* = 6) were selected, anesthetized by an intravenous injection of pentobarbital sodium (50 mg/kg BW), and then slaughtered for sample collection. Longissimus thoracis (LT) muscles were sampled, frozen in liquid nitrogen, and stored at −80°C for RNA extraction and chemical analysis. Furthermore, 1 cm^3^ LT sections between the 9th and 10th rib were frozen in liquid nitrogen for succinate dehydrogenase (SDH) staining or fixed with paraformaldehyde (PFA)/PBS (4%) for morphological analysis, respectively.

In Experiment 2, pigs from the control group and malic acid group (10 mg/kg) in Experiment 1 were fed the same three stages of diets for about 110 days until the finishing pigs were sacrificed. The three stages of diets were formulated based on the NRC (2012) recommendation for the nutrient requirements of 25–50 kg growing pigs, 50–75 kg growing pigs, and 75–100 kg finishing pigs, respectively. The ingredient composition and nutrient level of diets are shown in Supplementary Table 2. All pigs had free access to feed and water. Feed intake throughout the experimental period and the final BW were recorded. ADG, ADFI, and FCR were calculated as described in Experiment 1.

At the end of Experiment 2, pigs close to the average BW (~107 kg) of each group (*n* = 6) were humanly slaughtered by electrical stunning, exsanguinated, and eviscerated. LT muscles were rapidly sampled from the left-side carcass at the 10th rib and then frozen at −20°C for the analysis of amino acid and fatty acid composition.

### Carcass Traits

In Experiment 2, hot carcass weight of finishing pigs was recorded, and dressing percentage was calculated by dividing the hot carcass weight with the body weight. Values of backfat thickness opposite the thickest shoulder, the last rib, the 6th to the 7th rib, the 10th rib, and the last lumbar vertebra, as well as loin eye height and width [loin eye area (cm^2^) = loin eye height (cm) × width (cm) × 0.7] at the 10th rib were recorded. Fat-free lean index was estimated as follows: Fat-free lean index = 50.767 + [0.035 × hot carcass weight (Ib)] – [8.979 × the last rib fat thickness (in.)] (National Pork Producers Council, 1994).

### Meat Quality

Longissimus thoracis muscle samples on the left side of each carcass between the 10th and 12th ribs were collected in Experiment 2 for meat quality evaluation. Briefly, meat color score and marbling score of fresh meat were accessed using the National Pork Producer Council (NPPC) standards. Specially, for the meaning of meat color score, 1.0 is very pale, white and 6.0 is dark purplish red. At 45-min postmortem, a SPK pH meter (pH-star, DK2730, Herlev, Denmark) was used to measure initial pH_45min_ values of LT muscle. Meanwhile, at 24 h postmortem, pH_24h_ values were determined in a 4°C chilled room. Furthermore, the meat color parameters including lightness (L^*^), yellowness (b^*^), and redness (a^*^) were measured at 24-h postmortem using a tristimulus colorimeter (Minolta Chroma Meter Measuring Head CR-410 Minolta, Osaka, Japan) according to the standard method of CIE Lab system. The colorimeter was calibrated against a white tile according to the manufacturer's instruction.

Drip loss was measured as described previously (18), and calculated using the equation: drip loss (%) = [(initial weight – final weight)/initial weight] × 100. Additionally, a total of twelve muscle samples (about 100 g for each sample) were weighed and cooked in a water bath at 70°C for 30 min in one cooking batch. After being cooled to room temperature, the muscle sample was reweighed, and cooking loss was determined by calculating weight change percentage. Subsequently, shear force was tested using a digital display muscle tenderness meter (C-LM3B, Tenovo, Harbin, China) as described in our previous study (20). Each sample was measured with ten replicates.

About 50 g of each LT muscle sample was cut into thin slices (2–3 mm), weighed in aluminum boxes, and then put into a vacuum frozen dryer (Freezone 4.5™, Labconco Corp., Kansas City, MO, USA) for 72 h. Lyophilized muscle was subsequently crushed into powder and subsequently analyzed for intramuscular fat (IMF) content, amino acid composition, and fatty acid composition. IMF content was measured by Soxhlet petroleum–ether extraction (Budwi Extraction System B-11; Budwi, Lausanne, Switzerland) as previously described (21), and then converted to the weight percentage of fresh muscle weight.

### Texture Characteristics

After being cooked in a 70°C water bath for 30 min, LT samples in Experiment 2 were left for 2 h at room temperature and then cut into uniform cubes of ~1 cm^3^. Texture parameters including adhesiveness, springiness, cohesiveness, gumminess, chewiness, hardness, and shear force were measured by Texture Analyzer (TMS-Touch, Food Technology Corp., USA) with a probe P 0.5. The parameters were as follows: pretest speed, 0.5 mm/s; test speed, 1.0 mm/s; trigger type, auto-5 g; and strain, 50%.

### Amino Acids and Fatty Acids Composition of Skeletal Muscle

Amino acid concentration of LT samples in finishing pigs was determined based on the standard methods in AOAC (22). Specifically, concentrations of amino acids expect methionine, cysteine, and tryptophan, were determined by an amino acid analyzer (Hitachi L-8900, Tokyo, Japan) after hydrolysis with 6 mol/L HCl at 110°C for 24 h. Methionine and cysteine were determined by an amino acid analyzer (Hitachi L-8900, Tokyo, Japan) after cold performic acid oxidation overnight and hydrolyzing with 7.5 mol/L HCl at 110°C for 24 h. Tryptophan was determined by high performance liquid chromatography (Agilent 1200 Series, Santa Clara, CA, USA) after alkaline hydrolysis (LiOH) for 22 h at 110°C.

Fatty acid composition of LT muscle was determined by gas chromatography as described by AOAC (22) and our previous study (20). Briefly, 150 mg lyophilized muscle sample was extracted using 4 mL acetyl chloride methanol (acetyl chloride:methanol = 1:10), treated with 1 mL *n*-hexane and 1 mL internal standard FA solution (1 mg/mL C11:0) and kept in a 75°C water bath for 2 h. Then, the mixtures were blended with 5 mL potassium carbonate solution (70 g/L) and centrifuged at 900 r/min for 5 min. The supernatant was analyzed by gas chromatography (HP 6890 series, Hewlett Packard, Avondale, PA, USA), using a DB-23 capillary column (122–2,362; length 60 m, internal diameter 0.25 mm, film thickness 0.25 μm; Agilent Technologies Inc., Santa Clara, CA, USA) and a flame ionization detector. Fatty acid composition was presented as a milligram per 100 g muscle tissue basis.

### Serum and Skeletal Muscle Biochemical Parameters

The activities of superoxide dismutase (SOD), total antioxidant capacity (T-AOC), catalase (CAT), glutathione peroxidase (GSH-Px), and the content of malondialdehyde (MDA) in serum were determined by commercial kits (Nanjing Jiancheng Bioengineering Institute, Nanjing, China) to display the antioxidant capacity in Experiment 1. Furthermore, to analyze metabolic identifies of piglets in Experiment 1, concentration of glucose and lactate, and the activities of lactate dehydrogenase (LDH), MDH, and SDH in serum were measured. The actives of hexokinase (HK), LDH, MDH, and SDH in LT muscle were also determined by commercial kits (Nanjing Jiancheng Bioengineering Institute, Nanjing, China).

### Morphology of Skeletal Muscle

Longissimus thoracis muscle sections were dehydrated and embedded in paraffin after they were fixed in 4% PFA for 24 h. Specimens with thickness of 5 μm were sliced and then stained with hematoxylin and eosin. Images were captured with an Olympus CK40 microscope (Olympus Corporation, Japan). Muscle fiber crosssectional area (CSA) was determined using Adobe Photoshop (version CS6, United States).

### SDH Staining

Frozen muscle sections (10 μm) were incubated with SDH solution at 37°C for 30 min. After they were washed with distilled water two times, the sections were fixed with 4% PFA for 15 min, washed with distilled water, and incubated with 2% methyl green for 2 min, followed by careful washing. Sections were then sent for dehydration in alcohol and fixated by neutral balsam. Images were captured with an Olympus CK40 microscope (Olympus Corporation, Japan). Four different horizontal regions were captured in each section, and the amount of SDH staining fibers were analyzed.

### RNA Extraction and qRT-PCR Assays

Total RNA was extracted from LT muscles using RNAiso Plus (Takara, China) and reverse transcribed to cDNA by a PrimeScriptTM RT regent kit (Takara, China) according to the manufacturer's instruction. SybrGreen-based quantitative PCR was performed in a qTOWER 2.2 thermocycler (Analytik Jena, Jena, Germany) using β-actin as an internal control. The primers used in this study are presented in Supplementary Table 3. Relative gene expression was calculated by 2^−ΔΔ*Ct*^ method (23).

### Statistical Analysis

Data were presented as means ± SEMs, and analyzed by the unpaired two-tailed Student's *t*-test or the one-way ANOVA procedures of SAS (v.9.2, SAS Institute, USA). Linear and quadratic regression analysis were performed for evaluating the relationship between the growth performance and expository doses in Experiment 1. For growth performance data, each pen was treated as the experimental unit. Value of *p* < 0.05 was considered significant, and 0.05 ≤ *p* ≤ 0.10 was considered to have a trend.

## Results

### Growth Performance of Weaned Piglets

Growth performance of weaned piglets in Experiment 1 was summarized in Table 1. In detail, ADG, ADFI, and FCR were not affected by dietary supplementation of malic acid on day 14 compared with the control group. However, the regression analysis of ADFI and FCR presented a linear relationship from day 15 to day 28 and the overall period of the animal trial (0.05 ≤ *p* ≤ 0.10). Dietary supplementation of 10 mg/kg malic acid complex exhibited the highest values of ADFI (934 and 781 g, respectively), and the lowest values of FCR (0.46 and 0.51, respectively). Therefore, we focused on the role of malic acid in metabolic identifies and antioxidant capacity by employing the supplementation of 10 mg/kg malic acid complex in diets, named malic acid treatment in the subsequent study.

**Table 1:** Effects of dietary malic acid supplementation on the growth performance of weaned piglets (*n* = 6).

**Items**	**Control**	**Malic acid complex levels** **(mg/kg)**			**SEM**	***P*** **-value**		
		**2.5**	**5**	**10**		**ANOVA**	**Linear**	**Quadratic**
BW at d 0, kg	9.22	9.20	9.02	9.02	0.52	0.99	0.75	0.92
BW at d 28, kg	20.45	20.23	19.70	20.02	0.84	0.93	0.69	0.66
**d 1–14**
ADG, g	374	393	364	368	14	0.50	0.45	0.90
ADFI, g	540	616	587	615	50	0.67	0.40	0.42
FCR	0.70	0.66	0.65	0.60	0.04	0.58	0.18	0.89
**d 15–28**
ADG, g	428	395	399	418	16	0.46	0.92	0.14
ADFI, g	743	800	796	934	72	0.31	0.07	0.74
FCR	0.59	0.52	0.51	0.46	0.04	0.26	0.06	0.64
**d 1–28**
ADG, g	401	394	382	393	14	0.80	0.67	0.42
ADFI, g	639	713	691	781	57	0.36	0.10	0.99
FCR	0.64	0.58	0.57	0.51	0.04	0.26	0.06	0.72

### Aerobic Oxidation in Serum and Skeletal Muscle of Weaned Piglets

**Figure 1:**
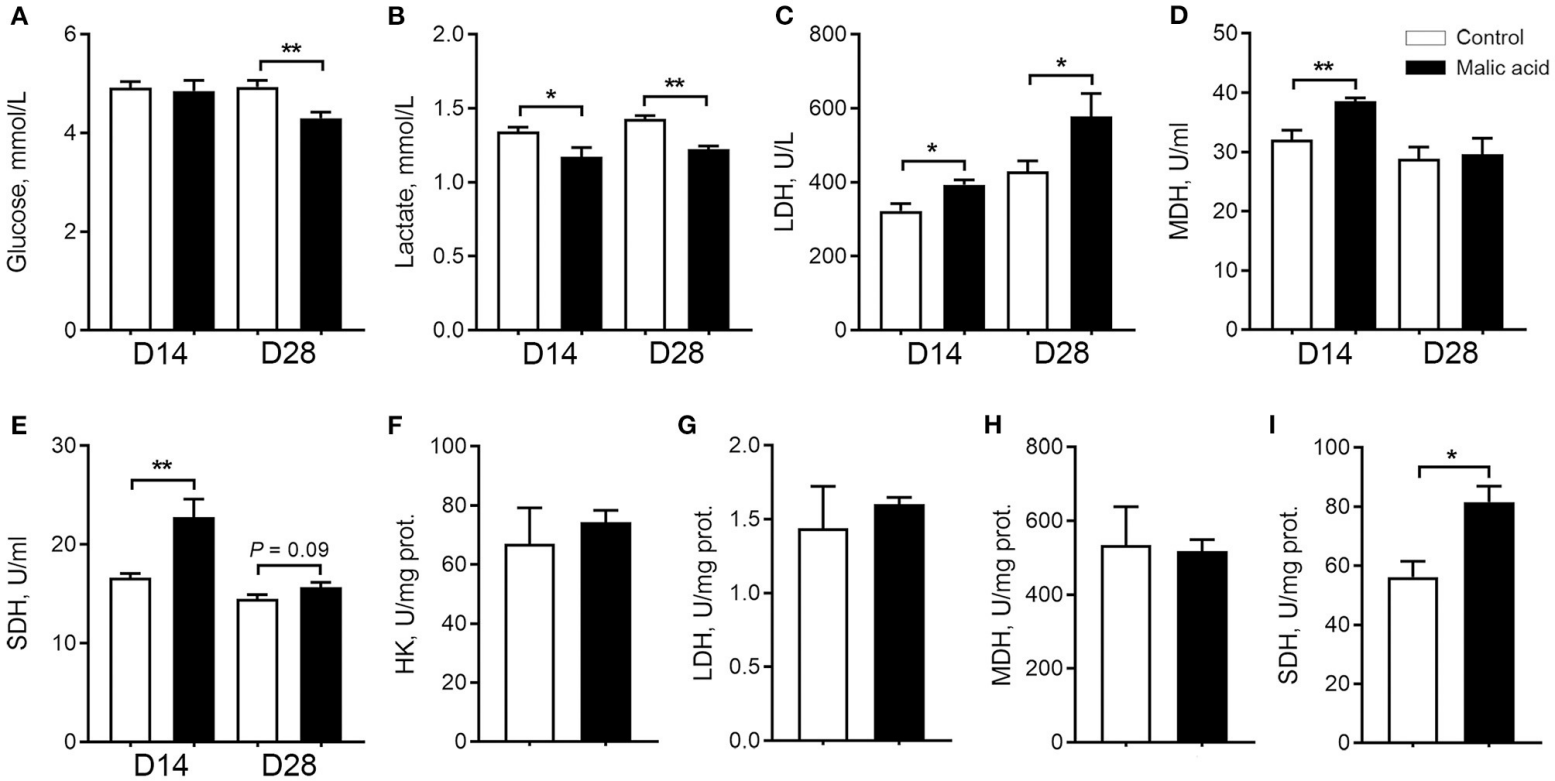
Effect of dietary malic acid supplementation on metabolic characteristics of weaned piglets. **(A,B)** Effects of malic acid on glucose and lactate levels in serum. **(C–E)** Effects of malic on LDH, MDH, and SDH activities in serum. **(F–I)** Effects of malic acid on activities of HK, LDH, MDH and SDH in skeletal muscle. D14 and D28 represented serum were collected on days 14 and 28 of the experiment. Data were presented as means ± SEM (*n* = 6). The statistical significance of difference between two means was calculated using *t*-test. *p < 0.05, **p < 0.01.

Here, we tested dietary malic acid supplementation on metabolism, especially aerobic oxidation in serum and skeletal muscle. We found that malic acid decreased circulating contents of glucose on day 28 (Figure 1A) and circulating contents of lactate on day 14 and day 28 (Figure 1B), suggesting that malic acid supplementation may be attributed to the elevated aerobic oxidation. Consistently, malic acid significantly enhanced the activity of MDH (Figure 1D) and SDH (Figure 1E) in serum on day 14. The activity of SDH also tended to increase upon malic acid treatment on day 28 (*p* = 0.09; Figure 1E), although the activity of LDH was also enhanced on day 14 and day 28 (Figure 1C). As for the metabolic status of skeletal muscle, the activity of SDH was also enhanced in malic acid group compared with that in control (*p* < 0.05; Figure 1I). Activities of HK, LDH, and MDH were not affected by malic acid supplementation (*p* > 0.05; Figures 1F–H).

### Skeletal Muscle Fiber-Type Transition in Weaned Piglets

**Figure 2:**
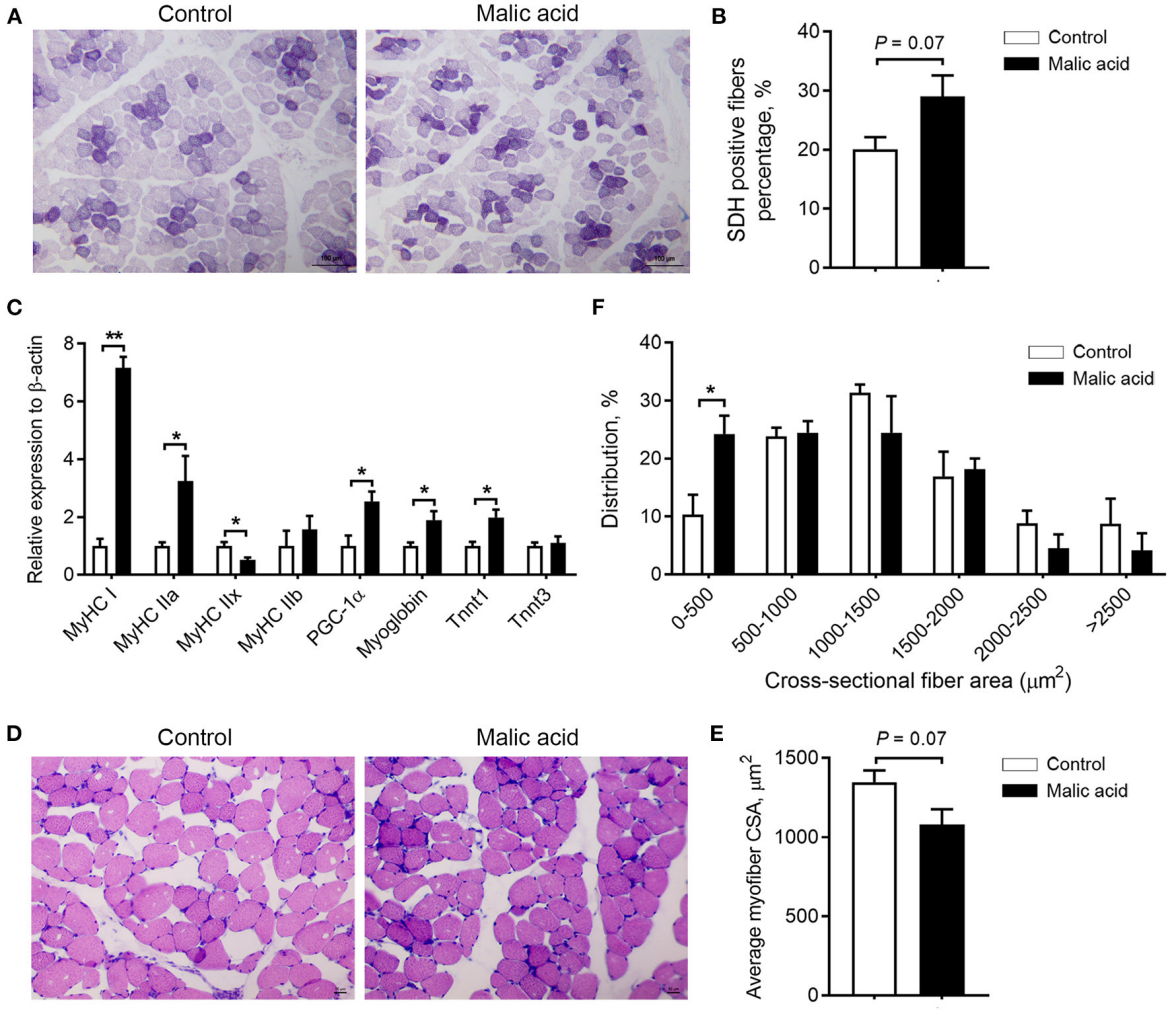
Effects of dietary malic acid supplementation on skeletal muscle fiber-type transition in LT muscles of weaned piglets. **(A,B)** Effects of malic acid on the percentage of SDH positive fibers. **(C)** Effects of malic acid on mRNA levels of *MyHCs, PGC1*α, *Myoglobin*, and *Tnnts*. **(D)** Reprehensive hematoxylin–eosin staining images of LT muscle. **(E)** Fiber average crosssectional area and **(F)** frequency histogram of fiber crosssectional area in **(D)**. Scale bars, 100 μm for **(A)** and 50 μm for **(D)**. Data are presented as means ± SEM (*n* = 6). The statistical significance of difference between two means was calculated using *t*-test. *p < 0.05, **p < 0.01.

In the present study, we studied the effects of malic acid on skeletal muscle fiber-type transition in LT muscle. SDH is a marker of oxidative capacity of skeletal muscle fibers. As exhibited by SDH staining, the percentage of SDH-positive fibers tended to increase after malic acid treatment relative to the control (*p* = 0.07; Figures 2A,B). Further, dietary malic acid supplementation also upregulated mRNA expression levels of oxidative fiber-associated genes *MyHC I, MyHC IIa*, PPARG coactivator 1 alpha (*PGC1*α), *Myoblogin*, and troponin T1 (*Tnnt1*), whereas it downregulated glycolytic fiber-associated genes *MyHC IIx*, although no change was observed in the expression level of *MyHC IIb* and troponin T3 (*Tnnt3*) (Figure 2C). Additionally, consistent with the switch of skeletal muscle fiber types, we found that malic acid tended to decrease the average CSA of muscle fibers (*p* = 0.07; Figures 2D,E) due to the increased proportion of small muscle fibers (0-500 μm^2^, *p* < 0.05; Figure 2F). This shift of muscle fiber size also indicated the contribution of malic acid to a switch toward oxidative fiber types.

### Antioxidant Capacity of Weaned Piglets

**Figure 3:**
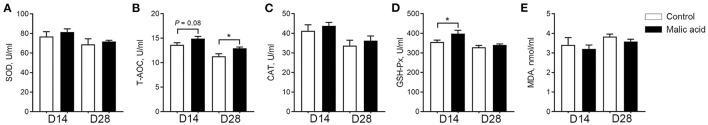
Effects of dietary malic acid supplementation on antioxidant capacity of weaned piglets. Activities of **(A)** SOD, **(B)** T-AOC, **(C)** CAT, **(D)** GSH-Px, and level of **(E)** MDA in serum on days 14 and 28 of the experiment. Data are presented as means ± SEM (*n* = 6). The statistical significance of difference between two means was calculated using *t*-test. *p < 0.05.

As shown in Figure 3, dietary malic acid supplementation induced the increase in the T-AOC activity of serum on day 14 (*p* = 0.08) and day 28 (*p* < 0.05), and GSH-Px activity of serum on day 14 (*p* < 0.05) when compared with the control group, whereas the activities of SOD and CAT, and the contents of MDA did not change.

### Growth Performance and Carcass Characteristics of Finishing Pigs

As shown in Supplementary Table 4, dietary malic acid supplementation in weaned piglets for 28 days exerted no effects on the index reflecting the subsequent growth performance of growing-finishing pigs, including final BW, ADG, ADFI and FCR. Furthermore, malic acid supplementation did not affect carcass weight, dressing percentage, lion eye area, backfat thickness, and fat-free lean index (*p* > 0.05).

### Meat Quality and Texture Characteristics

As shown in Table 2, there was no difference in meat quality traits, including pH_45min_, L^*^, a^*^, b^*^, cooking loss, shear force, marbling score, and intramuscular fat content among the control and malic-acid-supplemented pigs. Interestingly, dietary malic acid supplementation reduced drip loss significantly (*p* < 0.05) and tended to increase pH_24h_ (*p* = 0.07). Furthermore, dietary malic acid supplementation had no effects on texture characteristics of pork, including adhesiveness, springiness, cohesiveness, gumminess, chewiness, and hardness (Supplementary Table 5).

**Table 2:** Effects of dietary malic acid supplementation in weaned piglets on the meat quality of finishing pigs (*n* = 6).

**Items**	**Control**	**Malic acid**	**SEM**	***P*-value**
pH_45min_	6.11	6.13	0.13	0.34
pH_24h_	5.64	5.91	0.10	0.07
L* (lightness)	54.43	54.02	1.05	0.79
a* (redness)	19.21	18.73	0.20	0.13
b* (yellowness)	5.60	3.96	0.66	0.11
Drip loss, %	3.13	1.36	0.45	0.02
Cooking loss, %	31.86	30.74	1.55	0.62
Shear force, *N*	63.70	54.46	4.47	0.18
Marbling score (1–10 scale)	1.33	1.32	0.15	0.94
Intramuscular fat, %	2.14	1.78	0.36	0.51

### Muscle Amino Acid and Fatty Acid Composition of Finishing Pigs

As shown in Supplementary Table 6, compared to the control, dietary malic acid supplementation in weaned piglets had no effects (*p* > 0.05) on the content of essential amino acids (EAA), nonessential amino acids (NEAA), flavor amino acids, and the ratio of EAA/NEAA in the LD muscle of finishing pigs.

Fatty acid composition of total fat in LT muscle of finishing pigs was presented in Table 3. Briefly, the content of total saturated fatty acid (SFA) and contents of some SFA, such as C6:0, C8:0, C16:0, C18:0, and C20:0 were significantly decreased by the malic acid supplementation in weaned piglets (*p* < 0.05). Meanwhile, dietary supplementation of malic acid significantly decreased the content of total monounsaturated fatty acid (MUFA) and C18:1n9 (oleic acid) (*p* < 0.05), the largest proportion of fatty acids. Furthermore, the content of C18:3n3 was significantly decreased (*p* < 0.05) and the content of total polyunsaturated fatty acids (PUFA) tended to decrease (*P* = 0.09) when pigs were offered diets supplemented with 10 g/kg malic acid complex in their nursery period, whereas the content of C20:5n3 (eicosapentaenoic acid, EPA) was increased significantly (*p* < 0.01). The ratio of n-6 to n-3 PUFA was increased (*P* < 0.05) and ratio of PUFA to SFA tended to increase in the malic acid group (*P* = 0.05).

**Table 3:** Effects of dietary malic acid supplementation in weaned piglets on fatty acid composition of longissimus thoracis muscle of finishing pigs (mg/100 g muscle based on wet weight) (*n* = 6).

**Items**	**Control**	**Malic acid**	**SEM**	***P*-value**
SFA^a^	1,317.38	788.07	139.71	0.02
C6:0	0.72	0.93	0.04	<0.01
C8:0	0.51	0.34	0.02	<0.01
C10:0	3.41	2.06	0.42	0.05
C12:0	2.78	1.90	0.30	0.06
C14:0	40.33	25.48	5.16	0.07
C15:0	0.83	0.73	0.07	0.31
C16:0	767.75	458.07	79.92	0.02
C17:0	5.35	3.94	0.54	0.09
C18:0	472.14	278.30	52.37	0.03
C20:0	8.53	4.99	0.99	0.03
C21:0	11.82	7.78	1.41	0.07
C22:0	1.44	1.62	0.07	0.10
C23:0	0.53	0.55	0.03	0.75
C24:0	1.25	1.38	0.09	0.33
MUFA^b^	1,357.96	754.52	147.48	0.02
C14:1	0.39	0.32	0.07	0.52
C16:1	78.61	48.03	8.53	0.03
C18:1n9	1,251.39	689.91	137.30	0.02
C20:1	24.18	12.39	2.82	0.01
C22:1n9	1.74	1.67	0.13	0.70
C24:1	1.64	2.20	0.28	0.19
PUFA^c^	313.42	258.25	20.55	0.09
C18:2n6	253.89	196.53	20.00	0.07
C18:3n3	11.07	6.08	1.45	0.03
C20:3n6	4.98	5.01	0.17	0.90
C20:4n6	36.90	44.55	3.01	0.10
C20:3n3	2.66	1.65	0.32	0.05
C20:5n3 (EPA)	1.21	1.53	0.07	<0.01
C22:6n3 (DHA)	2.71	2.90	0.14	0.37
n-3 PUFA^d^	17.66	12.16	1.68	0.04
n-6 PUFA^e^	295.77	246.09	18.91	0.09
n-6/n-3	17.35	20.33	0.78	0.02
PUFA/SFA	0.24	0.39	0.05	0.05

a *SFA = C6:0 + C8:0 + C10:0 + C12:0 + C14:0 + C15:0 + C16:0 + C17:0 + C18:0 + C20:0 + C21:0 + C22:0 + C23:0 + C24:0*.

b *MUFA = C14:1 + C16:1 + C18:1n9 + C20:1 + C22:1n9 + C24:1*.

c *PUFA = C18:2n6 + C18:3n3 + C20:3n6 + C20:4n6 + C20:3n3 + C20:5n3 + C22:6n3*.

d *n-3 PUFA = C18:3n3 + C20:3n3 + C20:5n3 + C22:6n3*.

e *n-6 PUFA = C18:2n6 + C20:3n6 + C20:4n6*.

## Discussion

With the rapid development of swine industry, an increasing attention has been paid to the production of uniform and high-quality pork. Water holding capacity is a key quality attribute for pork products, which has an important impact on carcass yield, economic implications, and eating quality (2). The inextricable link among water holding capacity, meat color, pH values, tenderness, and other pork quality indicators has also been revealed in the previous study (9). Of note, formation of pork quality traits is dependent on the characteristics of the muscle. To be more specific, Berkshire pigs, which contained a significantly higher percentage of oxidative fibers than Landrace, Yorkshire, and crossbred pigs, showed the highest muscle pH values and lowest drip loss and lightness (24). Significant correlations were also observed between muscle fiber types in biopsied muscle and pork quality measured postmortem (25). Given that diets are important intrinsic factors to affect muscle characteristics directly (2), it necessitates the development of new dietary agents to improve pork quality, especially in the critical period of skeletal muscle fiber-type transition.

A number of studies have reported health-promoting effects of malic acid, such as modulating blood pressure by changing levels of l-arginine and NO (26) and enhancing the antioxidative defense system of aged rats (27). In consistent with studies performed in rats, dietary malic acid supplementation increased circulating actives of T-AOC and GSH-Px in our present work, indicating that malic acid enhanced antioxidant capacity of weaned piglets. In addition, malic acid is widely known as the intermediate of TCA cycle and plays an important role in transporting NADH from cytosol to mitochondria for energy production. In this study, dietary supplementation with malic acid resulted in the decreased level of blood glucose on day 28, and decreased level of blood lactate on days 14 and 28 of the experiment conducted in weaned piglets. The concomitant increase of SDH and MDH activities in serum were also detected on day 14. Loss of SDH would enhance succinate accumulation, block the TCA cycle, and reduce coupling efficiency of mitochondrial respiration to oxidative phosphorylation, leading to metabolic disorders (28, 29). Importantly, decreased SDH activity was observed accompanied with the microplastics-induced oxidative stress in reproductive system of male mice (30), and the increased SDH activity was beneficial to the antioxidant system of mung bean sprouts (31). Therefore, the improved antioxidant capacity of piglets may be partly attributed to the enhanced SDH activity in our study. As briefly mentioned above, these results undoubtedly demonstrated that malic acid promoted aerobic metabolism and ameliorated the health status of weaned piglets.

Metabolic adaptation is generally accompanied with endurance exercise and changes in skeletal muscle fiber types (32). In this study, oxidative capacity of skeletal muscle of weaned piglets was evaluated by SDH staining. Consistent with the dramatically enhanced SDH activity in the skeletal muscle, increased percentage of SDH-positive fibers suggested that the oxidative capacity of skeletal muscle was improved in malic acid group. Troponin T, the tropomyosin-binding protein, has three isoforms. *Tnnt1* and *Tnnt3* encodes slow and fast skeletal muscle troponin T, respectively (33). Combined with the increased expression level of *MyHC I, MyHC IIa, Myoglobin* and *Tnnt1*, our data demonstrated for the first time that dietary malic acid supplementation induced a switch from glycolytic to oxidative fibers. Although the exact mechanism behind the effects of malic acid was not known, it was proposed that increased mitochondrial biosynthesis or actives might be involved, evidenced by the increased expression level of PGC1α in skeletal muscle. PGC1α is regarded as a key master of mitochondrial biosynthesis and oxidative metabolism (34). Altered mitochondrial energy metabolism which resulted from the silencing or overexpression of PGC1α further underscored the role of this gene in energy homeostasis (35). Furthermore, increased PGC1α level and mitochondrial content were also required for succinate, another TCA cycle intermediate, to induce skeletal muscle fiber-type transition (36). Significantly elevated percentage of muscle fibers with smaller CSA was also observed coinciding with the muscle fiber-type transition, which may account for the adverse effect of malic acid on FCR of weaned piglets, although this effect was absent in the subsequent growth of growing–finishing pigs.

In the present study, the pH_24h_ and drip loss tended to increase and significantly decreased by malic acid supplementation during nursery period, respectively. Mechanically, high pH value and low drip loss were usually explained by the low postmortem glycolytic rate in oxidative fibers (37). Proteome analysis conducted in porcine semimembranosus and semitendinosus muscles further emphasized the close relationship between structural protein (slow type myosins and troponin complex), oxidative metabolic enzymes, and pork quality parameters (9). Similarly, lower serum lactate, higher serum and muscle SDH actives, and higher expression level of myosin 7 (*MyHC I*) and myosin 2 (*MyHC IIa*) were also induced by malic acid supplementation during the nursery period. However, future studies are needed to elucidate the direct connection between skeletal muscle remodeling induced by malic acid during nursery period and the improved pork quality.

Moreover, carcass traits, texture characteristics, and amino acid composition were not affected by dietary malic acid supplementation. Although intramuscular fat content was not changed, fatty acid composition of the LT muscle was modified. Briefly, the content of total SFA and total MUFA was significantly decreased by malic acid. Ameliorative effects of MUFA diets, such as improving insulin sensitivity (38) and reducing total mortality (39) have been reported. However, prospective cohort studies revealed that MUFA intake from animal sources was associated with higher mortality, although SFA in animal food may confound this association (40). Therefore, pork with lower MUFA content may contribute to an improvement of life quality. Importantly, the ratio of PUFA to SFA and the content of EPA were increased in malic acid group. Clinical studies and prospective cohort studies indicated that substitution of SFA and trans fatty acid for PUFA reduced the incidence of coronary heart disease (41, 42). Of significant importance was that n-3 PUFAs, especially EPA and docosahexenoic acid (DHA), were considered as potential adjuvant therapies for clinical management of COVID-19 patients due to their antiinflammatory and antioxidant effects (43). In this regard, dietary malic acid supplementation during the window period of muscle fiber-type transition could effectively improve the nutritional value of pork.

## Conclusions

The present study provided the first evidence that dietary malic acid supplementation during nursery period could enhance antioxidant capacity and increase the percentage of oxidative fibers of weaned piglets, and subsequently improve water holding capacity and nutritional values of pork, without negative effects on the growth performance of growing–finishing pigs. This work establishes a precise nutrition strategy to improve pork quality through inducing skeletal muscle fiber-type transition in the window period.

## Data Availability Statement

The original contributions presented in the study are included in the article/Supplementary Material, further inquiries can be directed to the corresponding author/s.

## Ethics Statement

The animal study was reviewed and approved by Animal Care and Use Committee of China Agricultural University.

## Author Contributions

XZ performed animal experiments, analyzed the data, and wrote the manuscript. JY and XZ contributed to the experimental design and manuscript modification. MC was mainly responsible for the sample collection, serum and skeletal muscle biochemistry, and qRT-PCR. EY, YW, CM, and PZ carried out meat quality test. All authors approved the manuscript.

## Funding

This work was supported by the National Key R&D Program of China (No. 2018YFD0500402).

## Conflict of Interest

The authors declare that the research was conducted in the absence of any commercial or financial relationships that could be construed as a potential conflict of interest.

## Publisher's Note

All claims expressed in this article are solely those of the authors and do not necessarily represent those of their affiliated organizations, or those of the publisher, the editors and the reviewers. Any product that may be evaluated in this article, or claim that may be made by its manufacturer, is not guaranteed or endorsed by the publisher.

## Abbreviations

ADFI: Average daily feed intake
ADG: Average daily gain
BW: Body weight
CAT: Catalase
CSA: Fiber crosssectional area
DHA: Docosahexenoic acid
EAA: Essential amino acids
EPA: Eicosapentaenoic acid
FCR: Feed conversion ratio
GSH-Px: Glutathione peroxidase
HK: Hexokinase
IMF: Intramuscular fat
LDH: Lactate dehydrogenase
LT: *Longissimus thoracis*
MDA: Malondialdehyde
MDH: Malate dehydrogenase
MUFA: Monounsaturated fatty acid
MyHC: Myosin heavy chain
NEAA: Non-essential amino acids
NPPC: National Pork Producer Council
NRC: National Research Council
PFA: Paraformaldehyde
PGC1α: PPARG coactivator 1 alpha
PUFA: Polyunsaturated fatty acid
SDH: Succinate dehydrogenase
SOD: Superoxide dismutase
T-AOC: Total antioxidant capacity
TCA: Tricarboxylic acid
Tnnt1: Troponin T1
Tnnt3: Troponin T3.
