# Supplementary material for: Dietary Malic Acid Supplementation Induces Skeletal Muscle Fiber-Type Transition of Weaned Piglets and Further Improves Meat Quality of Finishing Pigs
Source: Front Nutr. 2022 Jan 25;8:825495. doi: 10.3389/fnut.2021.825495 (PMC8821922; doi:10.3389/fnut.2021.825495)
Supplement: Supplementary file 1 [file Table_1.DOCX]

Table S1. Ingredient composition and nutrient content of basal diet in Experiment 1.

| Ingredient | Content, % | Nutrient levels^2^ | Content |
| --- | --- | --- | --- |
| Corn | 62.62 | Digestible energy, MJ/kg | 14.82 |
| Soybean meal | 10.00 | Crude protein, % | 18.10 |
| Extruded full-fat soybean | 10.00 | Calcium, % | 0.82 |
| Fish meal | 4.00 | Digestible phosphorus, % | 0.34 |
| Whey powder | 6.00 | SID lysine | 1.33 |
| Soy protein concentration | 2.00 | SID methionine + cysteine | 0.74 |
| Soybean oil | 1.75 | SID threonine | 0.80 |
| Dicalcium phosphate | 0.65 | SID tryptophan | 0.22 |
| Limestone | 1.09 |  |  |
| Salt | 0.30 |  |  |
| _L_-Lysine·HCl | 0.60 |  |  |
| _DL_-Methionine | 0.20 |  |  |
| _L_-Threonine | 0.23 |  |  |
| _L_-Tryptophan | 0.05 |  |  |
| Phytase | 0.01 |  |  |
| Vitamin-mineral premix^1^ | 0.50 |  |  |
| Total | 100.00 |  |  |

^1^ The premix provided the following per kg of diets: vitamin A, 10,000IU; vitamin D3, 1,500 IU; vitamin E, 30 IU; vitamin K3, 2.5 mg; vitamin B1, 1.5 mg; vitamin B2, 10 mg; vitamin B6, 10 mg; vitamin B12, 0.05 mg; folic acid, 1 mg; biotin, 0.5 mg; nicotinic acid, 30 mg; pantothenic acid, 20 mg; Cu, 20 mg; Fe, 100 mg; Zn, 110 mg; Mn, 40 mg; Se, 0.3 mg; I, 0.54 mg.

^2^ Data were calculated values.

Table S2. Ingredient composition and nutrient content of diets in Experiment 2.

| Items | Growing pigs |  |  | Finishing pigs |
| --- | --- | --- | --- | --- |
|  | 25-50 kg | 50-75 kg |  | 75-100 kg |
| Ingredient, % |  |  |  |  |
| Corn | 70.00 | 77.34 |  | 81.00 |
| Soybean meal | 20.60 | 15.50 |  | 11.66 |
| Wheat bran | 4.94 | 3.00 |  | 3.20 |
| Soybean oil | 1.00 | 1.00 |  | 1.20 |
| Limestone | 1.00 | 0.80 |  | 0.70 |
| Dicalcium phosphate | 1.00 | 0.90 |  | 0.90 |
| Salt | 0.20 | 0.20 |  | 0.20 |
| _L_-Lysine·HCl | 0.45 | 0.45 |  | 0.39 |
| _DL_-Methionine | 0.07 | 0.07 |  | 0.03 |
| _L_-Threonine | 0.13 | 0.13 |  | 0.11 |
| _L_-Tryptophan | 0.03 | 0.03 |  | 0.03 |
| 50% Choline chloride | 0.08 | 0.08 |  | 0.08 |
| Vitamin-mineral premix^1^ | 0.50 | 0.50 |  | 0.50 |
| Total | 100.00 | 100.00 |  | 100.00 |
| Nutrient levels^2^ |  |  |  |  |
| Digestible energy, MJ/kg | 14.27 | 14.35 |  | 14.35 |
| Crude protein, % | 15.71 | 13.84 |  | 12.46 |
| Calcium, % | 0.68 | 0.57 |  | 0.32 |
| Digestible phosphorus, % | 0.26 | 0.23 |  | 0.23 |
| SID lysine | 1.00 | 0.89 |  | 0.75 |
| SID methionine + cysteine | 0.54 | 0.50 |  | 0.43 |
| SID threonine | 0.60 | 0.53 |  | 0.47 |
| SID tryptophan | 0.18 | 0.15 |  | 0.14 |

^1^ The premix provided the following per kg of diets: vitamin A, 6,000 IU; vitamin D3, 2,400 IU; vitamin E, 20 IU; vitamin K3, 2 mg; vitamin B1, 0.96 mg; vitamin B2, 4 mg; vitamin B6, 2 mg; vitamin B12, 0.012 mg; folic acid, 0.4 mg; biotin, 0.04 mg; nicotinic acid, 22 mg; pantothenic acid, 11.2 mg; Cu, 120 mg; Fe, 76 mg; Zn, 76 mg; Mn, 12 mg; Se, 0.4 mg; I, 0.24 mg.

^2^ Data were calculated values.

Table S3. Primers used in real-time quantitative PCR

| Gene | Primer | Sequence (5’ → 3’) | GenBank ID |
| --- | --- | --- | --- |
| *MyHC I* | Forward | CGTGGACTACAACATCATAGGC | NC_010449 |
|  | Reverse | CTTTGCCCTTCTCAACAGGT |  |
| *MyHC IIa* | Forward | GGAGATCGACGACCTTGCTA | NM_214136 |
|  | Reverse | CTCCTTGGATTTCAGCTCGC |  |
| *MyHC IIx* | Forward | GAAACCGTCAAGGGTCTACG | NM_001104951 |
|  | Reverse | CGCTTCCTCAGCTTGTCTCT |  |
| *MyHC IIb* | Forward | GTTCTGAAGAGGGTGGTAC | NM_001123141 |
|  | Reverse | AGATGCGGATGCCCTCCA |  |
| *PGC-1α* | Forward | AGGGAAGAATACCGCAGAGA | NM_213963 |
|  | Reverse | TGTCCGTGTTGTGTCAGGTC |  |
| *Myoglobin* | Forward | GGAAGGTGGAGGCTGATGTC | NM_214236 |
|  | Reverse | CCGTGCTTCTTCAGGTCCTC |  |
| *Tnnt1* | Forward | TGAGGAAGGAAGAGGAAGAGGCTAAG | NM_213748 |
|  | Reverse | GTTTCATCTCACGCCCTGTCTGG |  |
| *Tnnt3* | Forward | CGAGACCCAAACTCACTGCT | NM_001001863 |
|  | Reverse | TCAGCCACGTTACTTCCACC |  |
| *β-actin* | Forward | TGCGGGACATCAAGGAGAAG | XM_021086047 |
|  | Reverse | AGTTGAAGGTGGTCTCGTGG |  |

Table S4. Effects of dietary malic acid supplementation in weaned piglets on the growth performance and carcass traits of finishing pigs (*n* = 6)

| Items | Control | Malic acid | SEM | *P* value |
| --- | --- | --- | --- | --- |
| Final body weight, kg | 107.87 | 106.60 | 4.85 | 0.86 |
| ADG, kg | 0.83 | 0.81 | 0.04 | 0.73 |
| ADFI, kg | 2.38 | 2.27 | 0.11 | 0.46 |
| FCR | 0.35 | 0.36 | 0.02 | 0.72 |
| Carcass weight, kg | 76.60 | 75.75 | 3.80 | 0.88 |
| Dressing percentage, % | 70.86 | 71.07 | 0.49 | 0.77 |
| Loin eye area, cm^2^ | 38.08 | 40.22 | 5.20 | 0.78 |
| Fat-free lean index, % | 52.48 | 51.46 | 0.58 | 0.25 |
| Subcutaneous backfat depth, cm |  |  |  |  |
| Shoulder fat thickness | 25.19 | 23.29 | 2.72 | 0.63 |
| The last rib fat thickness | 11.83 | 14.53 | 1.73 | 0.30 |
| The 6th to 7th rib fat thickness | 16.83 | 16.31 | 2.14 | 0.87 |
| The 10th rib fat thickness | 14.07 | 12.46 | 1.59 | 0.49 |
| Lumbosacral fat thickness | 6.49 | 8.69 | 0.94 | 0.13 |
| Average backfat depth | 13.87 | 15.06 | 1.99 | 0.68 |

Table S5. Effects of dietary malic acid supplementation in weaned piglets on the texture characteristics of finishing pigs (*n* = 6)

| Items | Control | Malic acid | SEM | *P* value |
| --- | --- | --- | --- | --- |
| Adhesiveness | 0.07 | 0.08 | < 0.01 | 0.33 |
| Springiness | 3.40 | 3.62 | 0.12 | 0.21 |
| Cohesiveness | 0.49 | 0.47 | 0.01 | 0.23 |
| Gumminess | 16.23 | 15.13 | 0.88 | 0.40 |
| Chewiness | 49.98 | 53.24 | 4.23 | 0.60 |
| Hardness, N | 32.61 | 31.83 | 1.53 | 0.73 |

Table S6. Effects of dietary malic acid supplementation in weaned piglets on amino acid composition of *longissimus thoracis* muscle of finishing pigs (% of dry meat weight) (*n* = 6)

| Items | Control | Malic acid | SEM | *P* value |
| --- | --- | --- | --- | --- |
| Essential amino acid (EAA) |  |  |  |  |
| Lysine (Lys) | 7.28 | 7.23 | 0.07 | 0.60 |
| Methionine (Met) | 2.15 | 2.21 | 0.04 | 0.31 |
| Threonine (Thr) | 3.77 | 3.74 | 0.04 | 0.57 |
| Tryptophan (Trp) | 1.05 | 1.08 | 0.01 | 0.11 |
| Valine (Val) | 3.88 | 3.86 | 0.04 | 0.65 |
| Leucine (Leu) | 6.65 | 6.60 | 0.06 | 0.54 |
| Isoleucine (Ile) | 3.81 | 3.84 | 0.04 | 0.68 |
| Arginine (Arg) | 5.05 | 5.09 | 0.07 | 0.70 |
| Histidine (His) | 3.83 | 3.95 | 0.12 | 0.49 |
| EAA^1^ | 40.85 | 40.96 | 0.35 | 0.83 |
| Non-essential amino acid (NEAA) |  |  |  |  |
| Asparagic acid (Asp) | 7.45 | 7.40 | 0.07 | 0.63 |
| Glutamic acid (Glu) | 11.51 | 11.46 | 0.12 | 0.79 |
| Glycine (Gly) | 3.46 | 3.46 | 0.09 | 0.99 |
| Alanine (Ala) | 4.42 | 4.45 | 0.06 | 0.79 |
| Proline (Pro) | 3.46 | 3.40 | 0.06 | 0.53 |
| Serine (Ser) | 3.05 | 3.03 | 0.04 | 0.64 |
| Tyrosine (Tyr) | 1.51 | 1.89 | 0.27 | 0.34 |
| Cysteine (Cys) | 0.96 | 0.97 | 0.02 | 0.58 |
| NEAA | 35.81 | 36.05 | 0.49 | 0.73 |
| EAA/NEAA^2^ (%) | 114.17 | 113.64 | 1.07 | 0.73 |
| Flavor amino acid^3^ | 31.89 | 31.85 | 0.37 | 0.95 |
| Total amino acid | 76.67 | 77.02 | 0.77 | 0.76 |

^1^ EAA = Lys + Met + Thr + Trp + Val + Leu + Ile + Arg + His

^2^ NEAA = Asp + Glu + Gly + Ala + Pro + Ser + Tyr + Cys

^3^ Flavor amino acid = Asp + Glu + Gly + Ala + Arg
